# Three-dimensional perfused tumour spheroid model for anti-cancer drug screening

**DOI:** 10.1007/s10529-016-2035-1

**Published:** 2016-05-11

**Authors:** Xiao Wan, Zhaohui Li, Hua Ye, Zhanfeng Cui

**Affiliations:** Department of Engineering Science, Institute of Biomedical Engineering, University of Oxford, Oxford, OX3 7DQ UK

**Keywords:** Anti-cancer drugs, Cancer spheroids, Drug screening, Perfusion, Three-dimensional cancer model

## Abstract

**Objective:**

To build an in vitro-perfused, three-dimensional (3D) spheroid model based on the TissueFlex system for anti-cancer drug efficacy testing in order to mimic avascular micro-tissues with inherent O_2_, nutrient and metabolite gradients, and to provide a more accurate prediction of drug toxicity and efficacy than traditional in vitro tumour models in conventional static culture well plates.

**Results:**

The perfused cancer spheroid model showed higher cell viability and increased diameter of spheroids over a relatively long culture period (17 days). Three anti-cancer drugs with different cytotoxic mechanisms were tested. In perfusion, lower cytotoxicity was observed for traditional cytotoxic drug 5-fluorouracil and microtubule-interfering, paclitaxel, showed greater interruption of spheroid integrity. For the hypoxic-dependent drug, tirapazamine, there was no significant difference observed between static and perfusion cultures.

**Conclusion:**

The perfusion culture provides a better homeostasis for cancer cell growth in a more controllable working platform for long-term drug testing.

**Electronic supplementary material:**

The online version of this article (doi:10.1007/s10529-016-2035-1) contains supplementary material, which is available to authorized users.

## Introduction

Three-dimensional (3D) cell-based in vitro models using tissue engineering is an emerging but promising technique for anti-cancer drug screening. Conventional drug testing relies on results obtained from animal-based in vivo tumour models, such as transplanting human cancer cells into immunodeficient mice, which has a high cost, a long duration of research and may give misleading results due to intrinsic interspecies differences (Nyga et al. 2011). To solve these problems, researchers have cultured cancer cells isolated from humans in 3D environments, which are known as 3D in vitro models. Compared with conventional cell-based in vitro models in two-dimensional (2D) monolayers, 3D in vitro models mimic the in vivo interactions between cells and the extracellular matrix (ECM) in studies on proliferation, differentiation, and gene expression of cancer cells (Bottaro et al. 2002).

Multicellular tumour spheroids (MCTS), which are the aggregates formed by cancer cells, are a widely applied 3D in vitro model. This model has potential to predict chemotherapy response profiles for clinical practice (Elliott and Yuan 2011). Techniques to create MCTS include liquid overlay, rotary vessel or spinner flask and a more recently-developed polymeric aqueous two-phase system (Atefi et al. 2014). One limitation of MCTS models is the limitation of spheroid growth over a long culture time: without blood perfusion, the passive diffusion of nutrient and O_2_ into the spheroids becomes insufficient to maintain the functions of the cells in the centre of the spheroids, which then causes significant necrosis when the spheroid diameter is >600 µm (Friedrich et al. 2009), and subsequently affects the accuracy of the following testing (Inamdar and Borenstein 2011). Here, we developed an in vitro 3D perfused cancer spheroid model for anti-cancer drug testing by using TissueFlex microbioreactors (Cui et al. 2007). MCTS were cultured in perfusion over 17 days, with increased growth limit compared to spheroids in conventional static culture. Three anti-cancer drugs with different mechanisms of action were tested to see if our system can reflect their characteristics: 5-fluorouracil is a pyrimidine analog interrupting cancer cell DNA replication (Longley et al. 2003), tirapazamine is activated to a toxic radical only in the hypoxic area of cancer tissues (Tung and Hsiao et al. 2011), and paclitaxel interferes with microtubule assembly in actively proliferating cells (Zhou and Giannakakou 2005).

## Methods and materials

### Cell line culture and maintenance

The colorectal cancer cell line, DLD-1, and the breast cancer cell line, NCI/ADR (ATCC), were cultured in high-glucose Dulbecco’s Modified Eagle Medium supplemented with 10 % (v/v) foetal bovine serum and 100 U penicillin/ml and 100 µg streptomycin/ml in a humidified incubator at 37 °C with 5 % CO_2_.

### TissueFlex microbioreactor production and spheroid formation

**Fig. 1:**
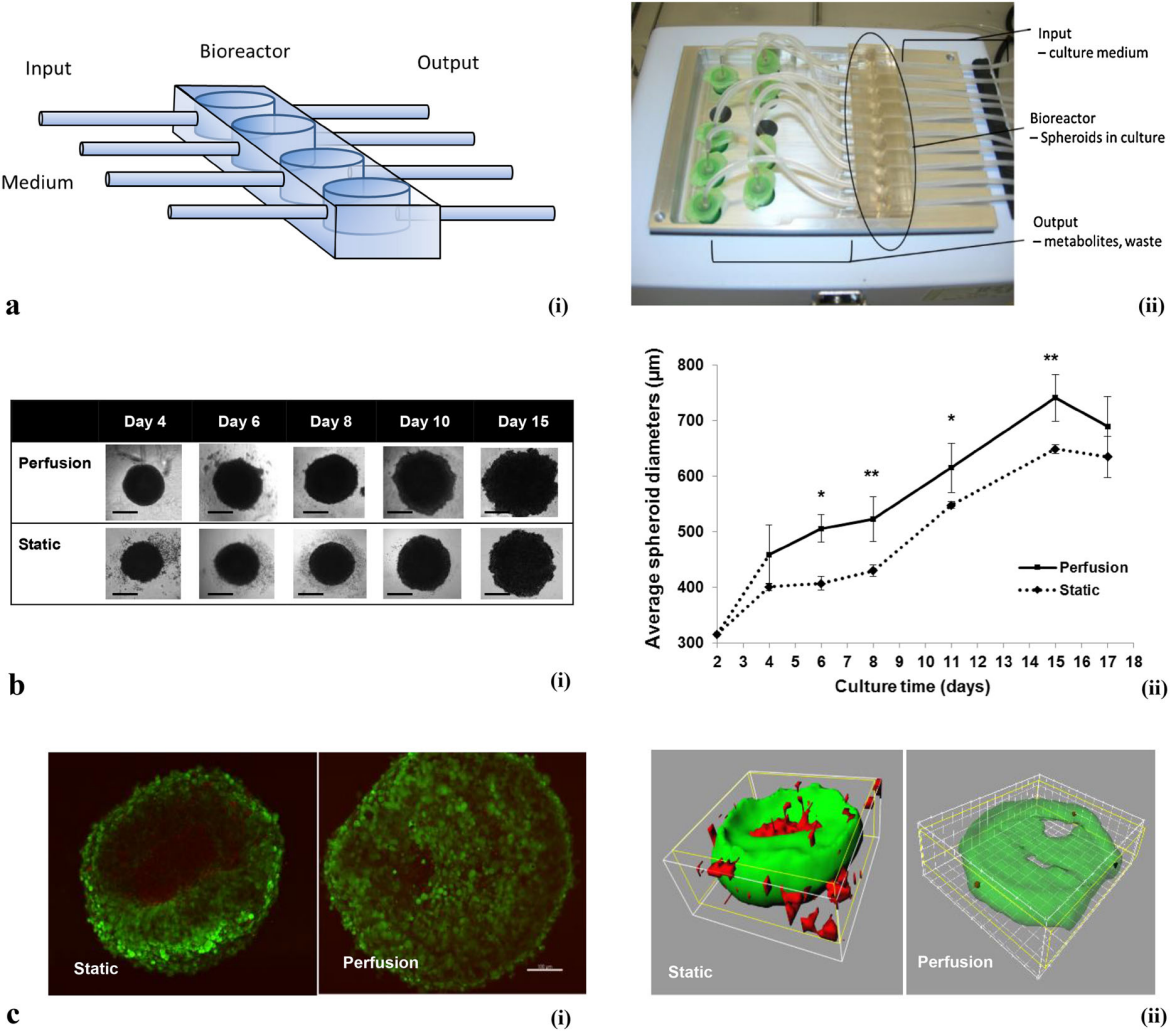
**a** (*i*) Schematic diagram of the bioreactor. The output is collected in eppendorf tubes and the input is controlled by mini syringe pumps. The well size is the same as those in normal 96-well plates. The transparent PDMS polymer allows the spheroids growth to be monitored real-time under an inverted microscope. (*ii*) Photo of the TissueFlex testing platform. **b** Growth kinetics of the spheroids. (*i*) Representative images of spheroids from Day 4 to Day 15. *Scale bar* 200 µm; (*ii*) Spheroid growth curve; between Day 6 and Day 15, significantly different diameters were observed between static and perfusion spheroids. *p < 0.05, **p < 0.01 by Student’s *t* test. **c** Multiphoton microscope imaging for live/dead staining. (*i*) Section imaging; (*ii*) 3D reconstruction of data set represented by section imaging in Fig. 1c-(i). *Scale bar* 100 µm

Poly(dimethylsiloxane) (PDMS) polymers and crosslinking reagent (Sylgard 184 silicone elastomer, Dow Corning) with the ratio of 10:1 were poured into a designed mould and cross-linked at 60 °C overnight. The product TissueFlex (Zyoxyel Ltd, UK), a single direction, multiple parallel perfused micro-bioreactor system is shown in Fig. 1a below. The nutrient rich culture media or tested anti-cancer drugs are individually perfused through inlet tubing by a syringe pump (Harvard Apparatus, US) to the bioreactors, where 3D micro-tissues are housed in the wells with the same size of the wells of 96-well plates, and the nutrient depleted medium is collected separately through the outlet tubing.

The surface of PDMS was treated with 0.1 M H_2_SO_4_ overnight at room temperature to make it hydrophilic, allowing the ensuring addition of agarose to form a concave surface. The wells were then rinsed with sterile distilled water three times, and 1.5 % (w/v) agarose was added to each well at 60 °C, and then allowed to solidify over 20 min at room temperature. Cell suspensions with 5 × 10^2^ to 3 × 10^3^ cells were added to each well, and the images of formed spheroids were captured using an inverted microscope (Nikon) and processed using software ImageJ (NIH, USA). The diameters of ten spheroids were measured and averaged for each group. Cells were seeded at 10^3^ well. (This is described in more detail in the supplementary information—Supplementary Fig. 1b).

### Cell viability analysis

Cell viability was assessed by AlamarBlue assay (Invitrogen) based on the manufacturer’s instructions. Briefly, AlamarBlue solution was incubated in each well for 2 h, then the fluorescence intensity at 560 nm excitation and 590 nm emission was measured using a micro-plate reader. Cell viability inhibition percentage = [(cell viability of untreated sample − cell viability of sample treated by anti-cancer drug)/cell viability of untreated sample] × 100 %.

### Cell live/dead imaging

A viability/cytotoxicity kit (Invitrogen) was used for imaging the live and dead cells. The red fluorescent ethidium homodimer-1 (EthD-1) only permeated through and stained the dead cells, while living cells allow the penetration of non-fluorescent acetomethoxy derivative of calcein (calcein AM) and degrade it into the green fluorescent calcein. The samples were visualised using a multi-photon microscope (MPM) (Zeiss, Germany). All serial optical sections from individual datasets were loaded into Imaris 7.6.1 (Bitplane Ag, Switzerland) software for processing and analysis.

### Perfusion and static culture

For perfusion culture, growth medium (DMEM with 10 % FBS), or DMEM containing drug or control (0.1 % DMSO) was perfused continuously from day 4 to day 17 at 10 µl/h using a 10-channel syringe pump (Harvard Apparatus, US). The same medium was renewed in static and monolayer cultures every 3 days from day 4 after seeding until day 17 (The cell seeding day was counted as day 1.) 10 µM 5-fluorouracil (5-FU) (Sigma), paclitaxel (Sigma) and tirapazamine (TPZ) (Toronto Research Chemicals Inc.) was tested, based on clinical drug dosage (Ando et al. 2008; Hong et al. 2011). Prior to drug exposure, spheroids were cultured without perfusion for 3 days in TissueFlex bioreactors or in 96-well plates (Friedrich et al. 2009).

### Data analysis

The data has been presented as the mean ± the standard error of the mean (SEM) of three independent experiments (n = 3). Two-tailed Student’s *t* test with *p < 0.05 was regarded as significantly different.

## Results and discussion

### Kinetics of spheroids growth in static and perfusion cultures

Figure 1b shows spheroids in both static and perfusion cultures that were allowed to grow until day 15. The average diameters of perfused spheroids were significantly larger than statically cultured spheroids from day 6 (at day 6 and 11, *p < 0.05; at day 8 and 15, **p < 0.01 by Student’s *t* test). In Fig. 1b, it is noticeable that the perfusion model increased the growth potential of the spheroids as shown by the spheroid diameter. At day 15 the average diameter of the spheroids in static culture reached 650 µm and had stopped growing. By contrast, in perfusion culture, the perfused spheroids reached an average diameter of 650 µm by day 12 and kept growing until the average diameter was 742 µm. A plateau in spheroid diameter was observed after day 15 in both static and perfusion cultures, and then the spheroid diameters decreased on day 17. It is possible that on day 15, the perfusion rate was not enough to provide nutrients to all the cells inside the spheroids. The cells in spheroids, especially in the central area of the spheroids, suffered from deficiency of nutrients so cells started to die and the number of viable cells started decreasing.

The dimensions of spheroid cultures bring challenges to conventional confocal microscopy, whose observable sample depth is ~100 µm. Figure 1c demonstrates how a multiphoton microscope (MPM) can solve this limitation in imaging spheroids, considered previously to be 1 mm (Graf and Boppart 2010). Figure 1c shows at day 15 there were significant distributions of dead cells in the central areas of spheroids in static culture, whereas only scattered dead cells were observed within the spheroids in perfusion culture. Figure 1c-ii is the 3D reconstruction image of Fig. 1c-i using Imaris software. The dead cells (shown in red) were observable in the static cultured spheroid, while the spheroid cultured in perfusion only showed scattered dead cells, revealed after the living cell areas (green) were viewed as semi-transparent.

In long-term static culture of 3D in vitro models, limited diffusion of nutrients causes dysfunction of cell organelles, especially for some highly metabolically active cell types such as hepatocytes (LeCluyse et al. 2012). The cumulated metabolites in medium induce oxidative stress and lead to the rapid loss of cell viability (Richert et al. 2006). In perfusion, O_2_ and nutrient supply are improved via pumping fresh medium into the system, and the tumour microenvironment homeostasis is maintained by removing metabolites continuously. In addition, perfusion culture prevents additional stress from sequential medium change and reduces the contamination risk (Wu and Kuo 2011).

### Anti-cancer drug testing

**Fig. 2:**
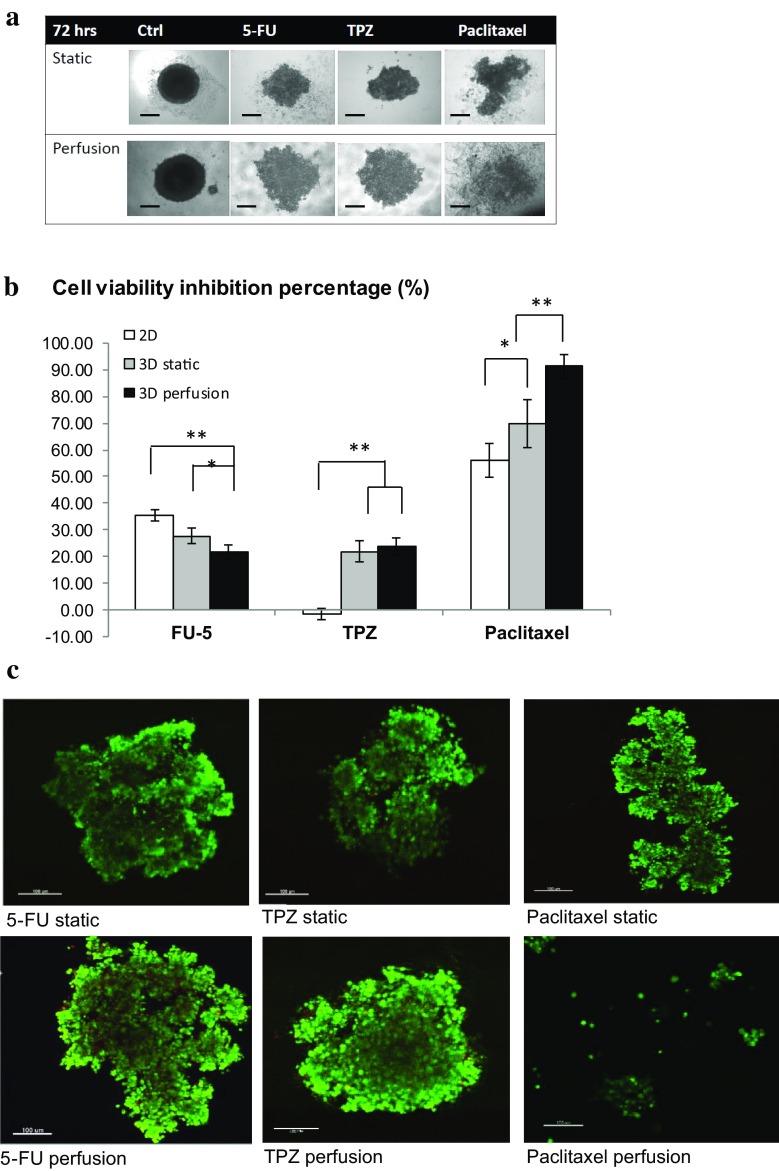
Drug testing comparing static and perfusion cultures. **a** Representative images of spheroids after 72 h of treatment with different drugs at the concentration of 10 µM. Note the significant morphological difference between static and perfused paclitaxel treatment. **b** Cell viability testing comparing perfusion and static spheroids. The drug concentration was 10 µM, after 72 h of treatment. A significant difference was observed between 3D static and perfusion testing of paclitaxel (p = 0.00312 by Student’s *t* test). **c** Representative MPM images of spheroids after treatment with different drugs. As shown in** a** and** c**, the spheroids perfused with paclitaxel totally lost the spheroid structures after 72 h. *Scale bar* 100 µm

The results in Fig. 2a suggest that there is significant morphological difference between static and perfused drug treatment, especially in the case of paclitaxel—in the static group there were still 3D structures remaining in the centre of the well, while in the perfused group the whole structure was nearly lost, with cell aggregates scattered over the well. Comparison of cell viability in Fig. 2b shows that after 72 h of 5-FU treatment, the highest cell viability inhibition was observed in the 2D monolayer model (35 %), and the 3D perfusion model showed the lowest drug response (21.7 %). A significant difference was observed between 2D and 3D perfusion cultures (p = 0.00706 by Student’s *t* test). 5-FU works as a pyrimidine analog to irreversibly inhibit thymidylate synthase and further interferes with cancer cell DNA replication; the effects of the drug clearly rely on it reaching the targeted cells (Longley et al. 2003). The larger size of spheroids in perfusion culture probably caused a lower drug penetration efficiency, due to the increased barriers for the drug penetration compared with 2D monolayers of cells.

For the tirapazamine (TPZ)-treated spheroids, significant drug inhibition was only observed in 3D models, with 21.8 % in static culture and 23.7 % in perfusion but there was no significant difference between the two 3D models. Unlike the cytotoxicity mechanism of 5-FU, the cytotoxicity of TPZ relies on the hypoxic central area of 3D structures so it is expected to have no effect in 2D monolayer cultures as all the cells are exposed to the same level of O_2_ (Tung and Hsiao et al. 2011).

Rather than killing cells directly like 5-FU, paclitaxel acts on proliferating cells by interfering with microtubule assembly. Microtubules are essential in tissue structure formation and morphology maintenance (Zhou and Giannakakou 2005). As shown in Fig. 2a and c, the spheroids statically treated by paclitaxel still had some 3D structures, while the spheroids perfused with paclitaxel totally lost the spheroid structures after 72 h with only small cell aggregates scattered in the well. The cytotoxicity of paclitaxel was more significant (p = 0.028 by Student’s *t* test) in 3D spheroids compared with 2D culture (56.2 %), and a significant difference (p = 0.00312 by Student’s *t* test) was observed between the viability inhibition percentages of spheroids in static (69.67 %) and perfusion cultures (91.3 %) as shown in Fig. 2b. How perfusion can enhance the cytotoxic effect of paclitaxel in spheroids needs to be further studied although it is possible that perfusion enhances the intercellular interactions and facilitates the anti-mitosis effect of paclitaxel.

## Conclusions

An in vitro 3D perfused spheroid model for anti-cancer drug testing was established. Spheroids perfused in TissueFlex showed improved cell viability and increased growth potential, compared with static cultured spheroids over 17 days of culture. The combination of multiphoton microscope imaging and perfusion enables detailed imaging. Perfusion provided precise control of the drug testing environment in a mid-throughput and parallel manner. In perfusion, paclitaxel showed its cytotoxicity more significantly compared with static culture, while 5-FU had less efficacy in perfusion spheroids compared with statically cultured ones. Future work will focus on more detailed study of spheroid functions and structures, and a high-throughput screening system will be developed on the basis of this work.

## Electronic supplementary material

Below is the link to the electronic supplementary material.

Supplementary material 1 (DOCX 146 kb)
