# Supplementary material for: Three-dimensional perfused tumour spheroid model for anti-cancer drug screening
Source: Biotechnol Lett. 2016 May 11;38:1389–95. doi: 10.1007/s10529-016-2035-1 (PMC4942491; doi:10.1007/s10529-016-2035-1)
Supplement: Supplementary file 1 — Supplementary material 1 (DOCX 146 kb) [file 10529_2016_2035_MOESM1_ESM.docx]

**Supplementary information**

*Multiphoton microscope parameters*

The samples were visualised using a multi-photon microscope (MPM) (Zeiss, Germany). Near infrared (NIR) laser beams (lambda = 800 nm) were obtained from a tunable 76 MHz femtosecond pulsed Ti: sapphire laser (Mira 900-F, Coherent, Ely, UK) pumped by a 10 W multiline argon ion laser (Verdi; Coherent). A Nikon S Fluor 10 × objective with a numerical aperture of 0.30 and working distance of 2.0mm was used for all images. The emission filters selected were 525 nm for green and 595 nm for red fluorescence.

*Optimisation of spheroid formation*

The breast cancer cell line NCI/ADR could only form small aggregates with uncontrollable sizes, while the colorectal cancer cell line DLD-1 was found to form one robust spheroid with repeatable diameters in the centre of each well (Fig. s1(a)). Gel strength of agarose also influenced the morphologies of spheroids. The gel strength of agarose 2576 is 400 g/cm^2^ at 1.5 % while agarose 9529 is much stronger at 1200 g/cm^2^. As a result, polymerised agarose 2576 (1.5 %, w/v) could not support cells to form an integral spheroid structure in the centre, while on gel formed by 1.5 % agarose 9539 (w/v) (Sigma-Aldrich, UK) cells formed firm spheroids. (b) Spheroid diameters formed by various initial cell seeding densities on day 4 in static culture only. After four days of culture, the diameters of the spheroids formed by DLD-1 at seeding densities between 5×10^3^ and 3×10^4^ cells/ml ranged from 174 to 861 µm. When the seeding density was 1×10^3^ cells in each well, the average diameter of the spheroids was 453 µm after four days of culture, and this was chosen as the seeding density for later growth kinetics and drug testing. The results are from three independent experiments.

**a
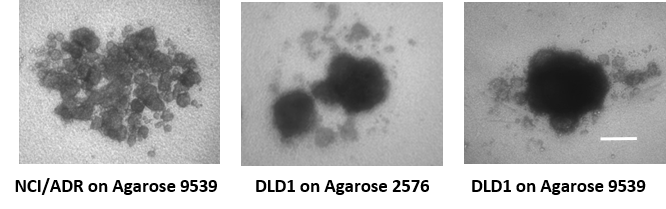
**

**b**
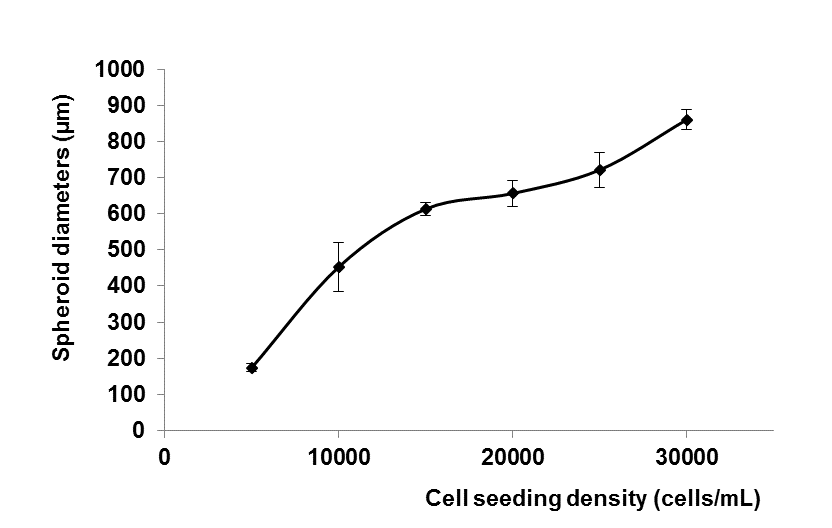


**Supplementary Fig. 1.** (a) Agarose type and cell type optimisation. From left to right: NCI/ADR on agarose 9539, DLD1 on agarose 2576, DLD1 on agarose 9539. (b) Initial cell seeding density optimisation on day 4. Seeding density of 10^3^ cells/well was chosen for the following experiments because the optimal initial diameter (~400 µm) was formed at this initial seeding density. *Scale bar = 200µm*
